# Case report: Orthostatic leg tremor as the initial manifestation in a patient with metabotropic glutamate receptor-5 encephalitis without cortical dysfunction: complexities in identification and treatment

**DOI:** 10.3389/fneur.2023.1288075

**Published:** 2023-12-15

**Authors:** Xia Yang, Qiong Liu, Ming-feng Lai, Xiao-hong Ma, Xiao-ting Hao, Jia-jun Xu, Wan-jun Guo

**Affiliations:** ^1^Mental Health Center, West China Hospital of Sichuan University, Chengdu, Sichuan, China; ^2^Psychiatric Laboratory and Mental Health Center, The State Key Laboratory of Biotherapy, West China Hospital of Sichuan University, Chengdu, Sichuan, China; ^3^Department of Neurology, West China Hospital, Sichuan University, Chengdu, China; ^4^Affiliated Mental Health Center and Hangzhou Seventh People's Hospital, Zhejiang University School of Medicine, Hangzhou Seventh People's Hospital, Hangzhou, Zhejiang, China

**Keywords:** mGluR5, autoimmune encephalitis, orthostatic leg tremor, prednisone, motor disorder

## Abstract

**Objective:**

Metabotropic glutamate receptor 5 (mGluR5) encephalitis is such a rare type of autoimmune encephalitis that its diagnosis remains a challenge.

**Case report:**

A 19-year-old female patient initially presented with anxiety and orthostatic leg tremors without cortical dysfunction. We found that this patient was positive for mGluR5 antibodies in both serum (1:1,000) and cerebrospinal fluid (1:32). After comprehensive intervention, the patient showed complete recovery at the 18-month follow-up.

**Discussion:**

This report expands our knowledge of the possible presentations of mGluR5 encephalitis for early diagnosis, which makes it possible to prevent serious consequences and improve the prognosis.

## Introduction

Autoimmune encephalitis (AE) is a group of novel neurological disorders associated with antibodies against neuronal cell-surface or synaptic proteins that can develop with abnormal symptoms in neurological and psychiatric manifestations (1). A recent epidemiological study estimated the prevalence rate of AE to be ~13.7/100,000, resulting in a heavy burden of disease with expensive costs (2). Since the discovery of the many common subtypes such as N-Methyl-D-Aspartate (NMDA), leucine-rich glioma inactivated (LGI1) (3, 4) and anti-Hu (3, 5) receptor encephalitis, an astonishing amount of novel AE antibodies have been described. Among the various novel AEs, metabotropic glutamate receptor (mGluR) encephalitis is so rare that it can present with a series of neuropsychiatric symptoms, particularly cortical dysfunction, but lacks specific symptoms and signs (all cases are summarized in Supplementary Table 1). Because reported cases are rare, clinicians cannot know about all the symptoms of mGluR5 encephalitis, let alone reach the same consensus on a diagnosis. For early diagnosis and treatment, clinicians, especially psychiatrists, should consider this disease while performing differential diagnoses. Here we report the case of a patient with anti-mGluR5 encephalitis who was initially admitted to a psychiatric ward for orthostatic leg tremor after being diagnosed with dissociative conversion disorder 2 months earlier.

## Case presentation

**Figure 1:**
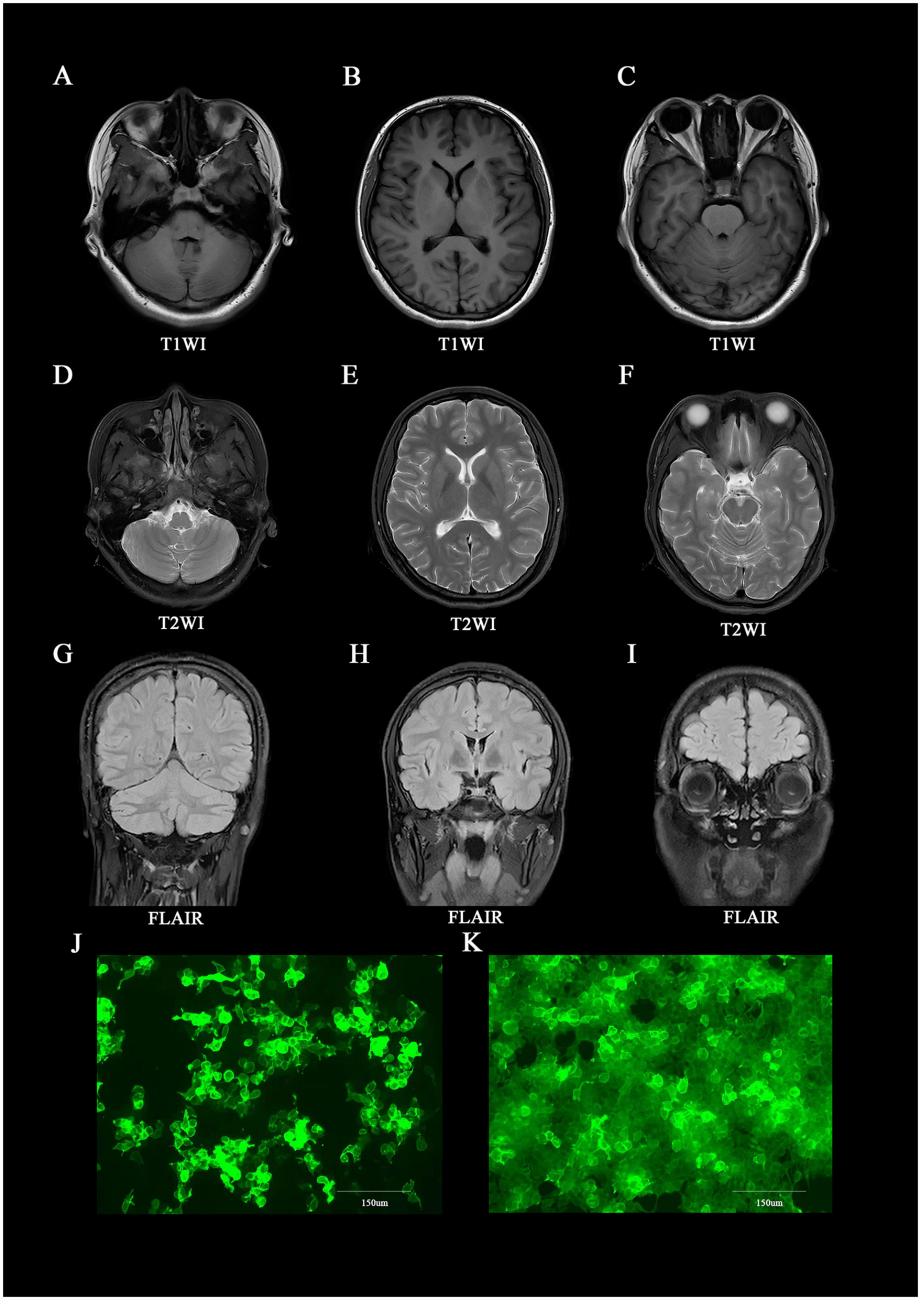
Brain MRI and immunofluorescence of anti-mGluR5 antibodies in the patient. **(A–I)** Brain MRI at the time of initial presentation, showing lesions on the whole of the brain. **(J, K)** Immunofluorescence against mGluR5 in **(J)** cerebrospinal fluid (1:32) and **(K)** serum (1:1,000).

A 19-year-old female patient (168 cm/77 kg), Han Chinese, a sophomore student, with a surgical history of left oophorectomy for teratoma 8 years earlier and a stressful event of failing an exam, was admitted to our hospital with anxiety, orthostatic leg tremor, sweating, and weight loss of 4 kg in 2 months. The outpatient doctor misdiagnosed her as having disjunctive-conversion disorder, and she received escitalopram oxalate 10 mg/d at the first treatment and was recommended to be hospitalized. The patient still had the above symptoms, and she could not complete Romberg's test or Straight-Line-Walking test when she was hospitalized for the disease course of 11 weeks. Repeat cranial MRIs showed no lesion in the brain (Figures 1A–I). Cancer biomarkers and immune indices in serum, electroencephalogram, and electromyography were negative (Supplementary material). The patient had a high level of IgG in the cerebrospinal fluid (CSF; 0.0749 g/L; normal range, 0.005–0.041 g/L). The cell count in the CSF was normal, and antibodies were detected against proteins related to autoimmune mGluR5 encephalitis (1:1,000 in serum and 1:32 in CSF; Figures 1J, K). Also, the oligoclonal band antibody was positive only in the CSF.

This patient was diagnosed with mGluR5 encephalitis and started on 5 days of high-dose intravenous methylprednisolone (1,000 mg/day) and intravenous immunoglobulins (0.4 g/kg/day). Subsequently, she received oral prednisone (45–55 mg/d) and anti-anxiety drugs (escitalopram oxalate, 10–20 mg/d) for 2 weeks. At 2 weeks after discharge (namely, a 2-week follow-up), the anxiety and orthostatic leg tremor were mildly alleviated (prednisone 35 mg/d and escitalopram oxalate 5 mg/d). Finally, the patient recovered completely at the 6-week follow-up (20 mg prednisone and 5 mg escitalopram oxalate), and her mGluR5-antibody titer in the serum was reduced to 1:32. Specific drug use is shown in the Supplementary Figure 2. The patient maintained recovery without any recurrence at the 18-month follow-up.

## Discussion

Given the finding of specific mGluR5 antibodies in both CSF and serum and the resolution of orthostatic leg tremors after immunotherapy therapy (immunoglobulin and steroids) and anti-anxiety drugs (escitalopram oxalate) in this patient, the diagnosis of mGluR5 encephalitis can be confirmed. Looking back at the course of the disease, this patient seemed to start with anxiety (psychiatric symptom) and gradually develop into orthostatic tremors, sweating, and weight loss. Psychiatric symptoms may be one of the potential and nonspecific early symptoms of mGluR5 encephalitis, but comorbidities with cognitive and motor disorders may have been more helpful in early identification. The following findings indicate that cognitive impairment may be more prevalent than motor disturbances in mGluR5 encephalitis. Based on a previous study in which 14 cases of mGluR5 encephalitis were enrolled, the clinical manifestations included mental and psychiatric disorders (13), cognitive disorders (11), sleep dysfunctions (9), seizures (8), and disorders of consciousness (6), but only four patients appeared to have motor disorders (6). Meanwhile, reviewing previous literature on motor disorders in mGluR5 encephalitis, we found that only one case reported postural hand tremor, and others showed orofacial dyskinesia, akinetic mutism, and psychomotor slowness, respectively (6, 7). In conclusion, although common symptoms of mGluR5 encephalitis may include psychotic symptoms, dysmnesia, or disorders of consciousness, clinicians should also pay attention to rare symptoms, for instance, motor symptoms (orthostatic leg tremor). Abnormal mGluR5 antibodies in the central nervous system may disrupt glutamate homeostasis and trigger the release of neurotoxic factors, which in turn may result in neurodegeneration in selective regions, including the cerebellum and amygdala (8–10). Some studies considered orthostatic tremor as the initial presenting feature in cerebellar and pontine lesions or autoimmune diseases (11, 12). In addition, sweating and weight loss could be prodromal symptoms in this case, which is consistent with previous research results (6).

In our case, the patient mainly manifested orthostatic leg tremors rather than mental and psychiatric symptoms or cognitive dysfunction. The possible reasons for the above phenomena may be that the patient was in the early stages of mGluR5 encephalitis or that there are some potential mechanisms of overlapping effects in the special trigger threshold of mGluR antibodies. Previous basic studies indicated that mGluR5 encephalitis classically presents with memory deficits and psychosis (7), and mGluR1 encephalitis may be mainly associated with cerebellar ataxia (13–15). However, mGluR1 and mGluR5 assemble into a homodimer with 85% similar amino acid sequence homologs in structure to activate subsequent receptors (14). We speculate that symptom presentation may overlap between mGluR1 and mGluR5 encephalitis at a specific threshold of triggering, for example, when the mGluR5-antibody titer in the serum and CSF is high.

A notable limitation is that in this case report, we were unable to perform bone marrow aspiration to exclude the possibility of cancer in the blood system. During the 18-month follow-up, we did not test the concentrations of mGluR5 antibodies in the CSF and only tracked the prognosis after 18 months.

## Conclusion

Although the motor complications of mGluR5 encephalitis may be rare, clinicians should recognize them as soon as possible, which will be helpful for early diagnosis and intervention.

## Data availability statement

The original contributions presented in the study are included in the article/Supplementary material, further inquiries can be directed to the corresponding authors.

## Ethics statement

Written informed consent was obtained from the individual(s) for the publication of any potentially identifiable images or data included in this article.

## Author contributions

XY: Writing – original draft. QL: Investigation, Writing – original draft. M-fL: Investigation, Writing – review & editing. X-hM: Writing – review & editing, Methodology. X-tH: Writing – review & editing, Conceptualization. J-jX: Writing – review & editing. W-jG: Conceptualization, Writing – review & editing.
