# Supplementary material for: Case report: Orthostatic leg tremor as the initial manifestation in a patient with metabotropic glutamate receptor-5 encephalitis without cortical dysfunction: complexities in identification and treatment
Source: Front Neurol. 2023 Dec 15;14:1288075. doi: 10.3389/fneur.2023.1288075 (PMC10755007; doi:10.3389/fneur.2023.1288075)
Supplement: Supplementary file 1 [file Table_1.DOCX]

**Table 1 Studies reviewed for mGluR5 encephalitis**

| **Num-ber** | **Gender (Age)** | **neuropsychiatric symptoms** | **prodromal symptom** | **Course** | **tumor** | **CSF** | **OB1** | **mGluR5 antibody** | **MRI/EEG** | **treatment** | **Prognosis** | **Follow-up time**(month) |
| --- | --- | --- | --- | --- | --- | --- | --- | --- | --- | --- | --- | --- |
| 1 Lancaster [1] | Female（46y） | Mental disorders; Cognitive disorders; movement disorders (myoclonic jerks) | Lymphadenopathy | 2 months | HL | WBC (+)  Protein (+) | NA | Serum: +  CSF: NA | MRI: +  EEG: NA | Steroids, Chemotherapy | Recovery | 48 |
| 2 Lancaster [1] | Male(15y) | Mental disorders; Cognitive disorders; Consciousness disorders; Seizures | Headache; nause | 5days | HL | WBC (+)  Protein (+)  IgG (+) | NA | Serum: NA  CSF: +++ | MRI: +  EEG: NA | Steroids | Recovery | 24 |
| 3 Mat [2] | Male (35y  ) | Mental disorders; Cognitive disorders | Lymphadenopathy; weight loss (9kg) | 6months | HL | WBC (+)  Protein (+)  IgG (+) | - | Serum: NA  CSF:+ (1:160) | MRI: +  EEG: + | Chemotherapy | Improved | 8 |
| 4 Prüss [3] | Female(30y) | Mental disorders; Cognitive disorders; Consciousness disorders; Seizures | Flu-like symptoms; weight loss (22kg) | 8months | (-) | WBC (+)  IgG (+) | NA | Serum:+ (1:1280)  CSF:+ (1:320) | MRI: -  EEG: + | Steroids, PE, RTX | Improved | 12 |
| 5 Guevara [4] | Male(68y) | Mental disorders; Cognitive disorders | Sweats; weight loss | 3months | HL | IgG (+) | + | mGluR5 + | EEG: -  MRI: + | Steroids, chemotherapy | Improved | 1 |
| 6 Spatola [5] | Female（46y） | Mental disorders; Cognitive disorders; movement disorders (myoclonic jerks) | Lymphadenopathy | 2 months | HL | WBC (+)  Protein (+) | NA | Serum: +  CSF: NA | MRI: +  EEG: NA | Steroids, Chemotherapy | Recovery | 48 |
| 7 Spatola [5] | Male (35y  ) | Mental disorders; Cognitive disorders | Lymphadenopathy; weight loss (9kg) | 6months | HL | WBC (+)  Protein (+)  IgG (+) | - | Serum: NA  CSF:+ (1:160) | MRI: +  EEG: + | Chemotherapy | Improved | 8 |
| 8 Spatola [5] | Female(30y) | Mental disorders; Cognitive disorders; Consciousness disorders; Seizures | Flu-like symptoms; weight loss (22kg) | 8months | (-) | WBC (+)  IgG (+) | NA | Serum:+ (1:1280)  CSF:+ (1:320) | MRI: -  EEG: + | Steroids, PE, RTX | Improved | 12 |
| 6 Spatola [5] | Male（75y） | Cognitive disorders; Progressive ophthalmoplegia; Dyskinesia (postural hand tremor, gait instability) | Weight loss (11kg) | NA | SCLC | WBC (+)  IgG (+) | NA | Serum:+ (1:160)  CSF:+ (1:320) | MRI：+ | Steroids, Chemotherapy | Improved | 62 |
| 7 Spatola [5] | Female（40y） | Mental disorders; Cognitive disorders; Consciousness disorders; Insomnia; Orofacial dyskinesia | Headache | NA | (-) | WBC (+)  IgG (+) | NA | Serum:+ (1:1280)  CSF:NA | MRI: -  EEG：NA | Steroids, PE | Recovery | 20 |
| 8 Spatola [5] | Male（16y） | Mental disorders; Consciousness disorders; Insomnia; Dystonia; Seizures | Headache | NA | HL | WBC (+)  IgG1/G3 (+) | + | Serum:+ (1:1280)  CSF:+ (1:20) | MRI：-EEG：NA | Steroids, chemotherapy, PE | Recovery | 48 |
| 9 Spatola [5] | Female（6y） | Cognitive disorders; Consciousness disorders; Insomnia; Dyskinesia; Seizures | Rash, headache, flulike symptoms, | NA | (-) | WBC (+)  IgG1 (+) | - | Serum:NA  CSF:+ (1:10) | MRI: + | Steroids,  RTX | Improved | 19 |
| 10 Spatola [5] | Female（20y） | Mental disorders; Cognitive disorders; Consciousness disorders | Headache, flu-like symptoms | NA | (-) | WBC (+)  IgG1/G2 (+) | + | Serum:+ (1:1280)  CSF:NA | MRI：-EEG：NA | None | Recovery | 96 |
| 11 Spatola [5] | Male（15y） | Mental disorders; Cognitive disorders; Insomnia; Facial paralysis | *NA* | NA | HL | WBC (+)  IgG1/G2/G3 (+) | + | Serum:+ (1:1280)  CSF:+ (1:640) | MRI：-  EEG：NA | Steroids, chemotherapy | Improved | 12 |
| 12 Spatola [5] | Male（49y） | Mental disorders; Consciousness disorders; Seizures; Insomnia | *NA* | NA | (-) | WBC (+) | + | Serum:+ (1:320)  CSF:+ (1:160) | MRI: -  EEG：NA | Steroids | Improved | 5 |
| 13 Chen[6] | Male  (15y) | Mental disorders; Seizures | *NA* | *NA* | (-) | *NA* NMDA (+) | *NA* | NA | MRI: -  EEG: + | IVIg, Steroids, Chemotherapy | Improved | 3 |
| 14 Guo[7] | Male  (32y) | Mental disorders; Cognitive disorders; Insomnia | fever, headache | 8 months | (-) | IgG (+) | *NA* | Serum:+ (1:320)  CSF:+ (1:3.2) | MRI: -  EEG: *NA* | IVIg, Steroids, Chemotherapy | Recovery | 24 |
| 15 Liu[8] | Female  (12y) | Consciousness disorders; Seizures | *NA* | 20 days | (-) | IgG (+)  IgM (+) | + | Serum:NA  CSF:+ (1:32) | MRI: -  EEG: + | IVIg, Steroids | Recovery | 3 |
| 16 Chen[9] | Male  (51y) | Mental disorders; Seizures | fever; hidrosis | 2 months | (-) | WBC (+)  IgG (+) | *NA* | Serum:+ (1:10)  CSF:+ (1:32) | MRI: -  EEG: + | IVIg, Steroids, RTX | Died | *NA* |
| 17 Feng[10] | Female  (22y) | Insomnia; Paroxysmal abduction of right limb in sleeping | Fall | 2 months | (-) | WBC (+) | - | Serum:+ (1:32)  CSF:+ (1:1) | MRI: -  EEG: + | IVIg, chemotherapy | Improve | 6 |
| 18 Zhang[11] | Female  (22y) | Consciousness disorders; Cognitive disorders; Ptosis of eyelids | double vision; weight loss | 1 month | (-) | WBC (+) | + | Serum:+ (1:32)  CSF:+ (1:1) | MRI: +  EEG: - | Steroids | Improve | 2 |
| 19 Yang[12] | Male  (44y) | Blurred vision; slurred speech; dysphagia; dysarthria; weakened muscle strength, numbness | Headache; nausea; vomiting, | 4 days |  | Protein (+) | + | Serum:+ (1:30)  CSF:+ (1:1) | MRI: -  EEG: - | IVIg | Recovery | 6 |
| 20 this case | Female  (19y) | Anxiety, postural tremor | Hidrosis; weight loss | 1month | (-) | IgG (+) | CSF: +;  Serum: - | Serum:+ (1:1000)  CSF:+ (1:32) | MRI: -  EEG: - | Steroids, IVIg | Recovery | 6 |
| **HL**: Hodgkin Lymphoma; **SCLC**: Small - cell lung cancer; **PE**: plasma exchange; **RTX**: rituximab; **IVIg**: IV immunoglobulin | | | | | | | | | | | | |

Conference:

1. Lancaster, E., et al., *Antibodies to metabotropic glutamate receptor 5 in the Ophelia syndrome.* Neurology, 2011. **77**(18): p. 1698-701.

2. Mat, A., et al., *Ophelia syndrome with metabotropic glutamate receptor 5 antibodies in CSF.* Neurology, 2013. **80**(14): p. 1349-50.

3. Prüss, H., et al., *Limbic encephalitis with mGluR5 antibodies and immunotherapy-responsive prosopagnosia.* Neurology, 2014. **83**(15): p. 1384-6.

4. Guevara, C., et al., *Encephalitis Associated to Metabotropic Glutamate Receptor 5 (mGluR5) Antibodies in Cerebrospinal Fluid.* Front Immunol, 2018. **9**: p. 2568.

5. Spatola, M., et al., *Encephalitis with mGluR5 antibodies: Symptoms and antibody effects.* Neurology, 2018. **90**(22): p. e1964-e1972.

6. Chen, J., et al., *Single or multiple positive anti-neuronal antibodies to autoimmune Clinical analysis of encephalitis.* Modern practical medicine, 2020. **32**(07): p. 774-776.

7. Guo, K.-d., j.-f. Lin, and Z. Hong, *A case of anti-mGluR5 encephalitis.* Chin J Nerv Ment Dis, 2021(01): p. 44-47.

8. Liu, Y.-b., Y.-c. Chen, and x.-y. Wu, *mGluR5 autoimmune encephalitis in children: A case report and literature review.* Chin J Clin Neurosci, 2021. **29**(06): p. 680-685.

9. Chen, Y.-q., H. Gu, and L.-g. Xu, *A case report of anti-metabotropic glutamate receptor 5 encehpalitis with extreme delta brush in electroencephalogram.* Chin J Clin Neurosci, 2021(02): p. 131-135.

10. Feng, J.-l., et al., *Clinical analysis of 14 patients with anti-metabotropic glutamate receptor 5 encephalitis.* Chin J Neuromed, 2022(02): p. 172-175.

11. Feng, J.-l., et al., *Autoimmune encephalitis associated with anti-metabotropic glutammate receptor-5 antibody: A case report.* Chin J Clin Neurosci, 2022(02): p. 172-175.

12. Yan, W., et al., *Case Report: Guillain-Barré Syndrome Characterized by Severe Headache Associated With Metabotropic Glutamate Receptor 5 Antibody.* Front Immunol, 2022. **13**: p. 808131.

8. Lancaster E, et al.，*Antibodies to metabotropic glutamate receptor 5 in the Ophelia syndrome. Neurology. 2011 Nov 1;77(18):1698-701.*

9. Mat A, et al.，*Ophelia syndrome with metabotropic glutamate receptor 5 antibodies in CSF. Neurology. 2013 Apr 2;80(14):1349-50.*

10 Prüss H, et al.，*Limbic encephalitis with mGluR5 antibodies and immunotherapy-responsive prosopagnosia. Neurology. 2014 Oct 7;83(15):1384-6.*

11 Guevara C, et al.，*Encephalitis Associated to Metabotropic Glutamate Receptor 5 (mGluR5) Antibodies in Cerebrospinal Fluid. Front Immunol. 2018 Nov 5;9:2568.*

12 Spatola M, et al.，*Encephalitis with mGluR5 antibodies: Symptoms and antibody effects. Neurology. 2018 May 29;90(22):e1964-e1972.*
