# Supplementary material for: Case report: Orthostatic leg tremor as the initial manifestation in a patient with metabotropic glutamate receptor-5 encephalitis without cortical dysfunction: complexities in identification and treatment
Source: Front Neurol. 2023 Dec 15;14:1288075. doi: 10.3389/fneur.2023.1288075 (PMC10755007; doi:10.3389/fneur.2023.1288075)
Supplement: Supplementary file 2 [file Table_2.DOCX]

**Supplemental Materials**

**Postural tremor symptom as initial manifestation in a patient of metabotropic glutamate receptor-5 encephalitis without cortical dysfunction: complexities in identification and treatment**

**Supplemental Text**

Other **antibodies of encephalitis** (NMDAR, AMPA1, AMPA2, GABABR, LGI1, CASPR2, DPPX, IgLON5, GlyRα1, GABAAR, D2R, Neurexin-3α, KCNA4), **paraneoplastis** (Hu, Yo, Ri, CV2, Amphiphysin, Ma1, Ma2, OX1, Tr, Zic4, GAD65, Titin, Recoverin, PKCr) and **cerebellitis** (Homer3, ATP1A3, ARHGAP26, ITPR1, Septin5, NCDN, Hu, Yo, Ri, CV2, Ma2, Amphiphysin, Tr, Zic4, Ma1, GAD, PKCr, SOX1) **were negative both in serum and CSF**.

**Supplementary table 1. Results of immune globulin and autoimmune antibodies in serum**

| items | results | Reference criteria |
| --- | --- | --- |
| IgG | 27 g/L ↑ | 8-15.5 g/L |
| IgA | 2670 mg/L | 836-2900 mg/L |
| IgM | 1080 mg/L | 700-2200 mg/L |
| IgE | 157 IU/mL ↑ | 5-150 IU/mL |
| C3 | 0.646 g/L ↓ | 0.785-1.52 g/L |
| C4 | 0.0672 g/L ↓ | 0.145-0.36 g/L |
| RF | <20 IU/mL | <20 IU/mL |
| PFB | 186 mg/L ↓ | 190-500 mg/L |
| ANA | +/- | - |
| dsDNA | 3.07 IU/mL | <30 IU/mL |
| U1-Nrnp/Sm | - | - |
| Sm | - | - |
| SS-A | +/- | - |
| Ro-52 | **+** ↑ | - |
| SS-B | - | - |
| Scl-70 | - | - |
| PM-Scl | - | - |
| Jo-1 | - | - |
| CENP B | - | - |
| PCNA | - | - |
| ANuA | - | - |
| AHA | - | - |
| ARPA | - | - |

**Ig**: immune globulin; **RF**: rheumatoid factors; **PFB**: properdin factor B; **ANA**: antinuclear antibody; **dsDNA**: anti-dsDNA antibodies; **U1-Nrnp/Sm**: anti-U1-nRNP/smith antibodies; **SS-A**: anti-SS-A antibodies; **SS-B**: anti-SS-B antibodies; **Scl-70**: anti- Scl-70 antibodies; **PM-Scl**: anti- PM-Scl antibodies; **Jo-1**: anti- Jo-1 antibodies; **CENP B**: anti- CENP B antibodies; **ANuA**: anti-nucleosome antibodies; **AHA**: anti-histone antibody; **ARPA**: Anti- ribosomal P protein antibodies.

**Supplementary table 2. The electrophysiological tests included check of nerve conduct function of both sensation and motor at four limbs**

|  | Lat/SD (ms) | Amp/SD (mV) | CV/SD (m/s) | Amp %/SD (%) | F/SD (ms) |
| --- | --- | --- | --- | --- | --- |
| **MOTOR NERVES** | |  |  |  |  |
| Right Medianus |  |  | 63.5 | -3 | 22.7 |
| Wrist-APB | 2.7 | 9.1 |  |  |  |
| Bel Elb-Wrist | 6.4 | 8.8 |  |  |  |
| Left Medianus |  |  | 62.9 | -6 | 22.3 |
| Wrist-APB | 2.3 | 9.0 |  |  |  |
| Bel Elb-Wrist | 5.8 | 8.5 |  |  |  |
| Right Ulnaris |  |  | 66.8 | -11 | 23.5 |
| Wrist-ADM | 1.83 | 14.2 |  |  |  |
| Bel Elb-Wrist | 5.8 | 12.7 |  |  |  |
| Left Ulnaris |  |  | 64.4 | 2 | 23.5 |
| Wrist-ADM | 1.92 | 13.3 |  |  |  |
| Bel Elb-Wrist | 5.8 | 13.6 |  |  |  |
| Right Tibialis |  |  |  |  | 46.5 |
| Ankle-AHB | 3.3 | 37.2 |  |  |  |
| Left Tibialis |  |  |  |  | 42.7 |
| Ankle-AHB | 2.2 | 31.0 |  |  |  |
| Right Peroneus |  |  | 46.1 | 7 | 45 |
| Ankle-EDB | 3.2 | 6.1 |  |  |  |
| Be knee-Ankle | 10.8 | 6.6 |  |  |  |
| Left Peroneus |  |  | 47.4 | 2 | 46 |
| Ankle-EDB | 2.5 | 11.4 |  |  |  |
| Be knee-Ankle | 10.2 | 11.6 |  |  |  |
| **SENSORY NERVES** | |  |  |  |  |
| Right Medianus |  |  |  |  |  |
| Dig III-Wrist | 1.92/0.4 | 34 | 65.1 |  |  |
| Left Medianus |  |  |  |  |  |
| Dig III-Wrist | 2.0/0.9 | 39 | 67.5 |  |  |
| Right Ulnaris |  |  |  |  |  |
| Dig V-Wrist | 1.56/-3.2 | 16 | 64.1 |  |  |
| Left Ulnaris |  |  |  |  |  |
| Dig V-Wrist | 1.67/-2.9 | 15 | 65.9 |  |  |
| Right Suralis |  |  |  |  |  |
| Stim 2-Rec 2 | 2.1 | 19 | 52.4 |  |  |
| Left Suralis |  |  |  |  |  |
| Ankle-Foreleg | 1.85 | 26 | 56.8 |  |  |

|  | Amp |  | Dur |  | Area | Poly% |
| --- | --- | --- | --- | --- | --- | --- |
| MUSLE | uV | rel. SD | ms | rel. SD | uVms |  |
| Right Tibialis anterior | 600 | 0.2 | 11.1 | -0.2 | 1380 | 46 |
| Left Tibialis anterior | 775 | 1.2 | 11.2 | -0.2 | 1029 | 21 |


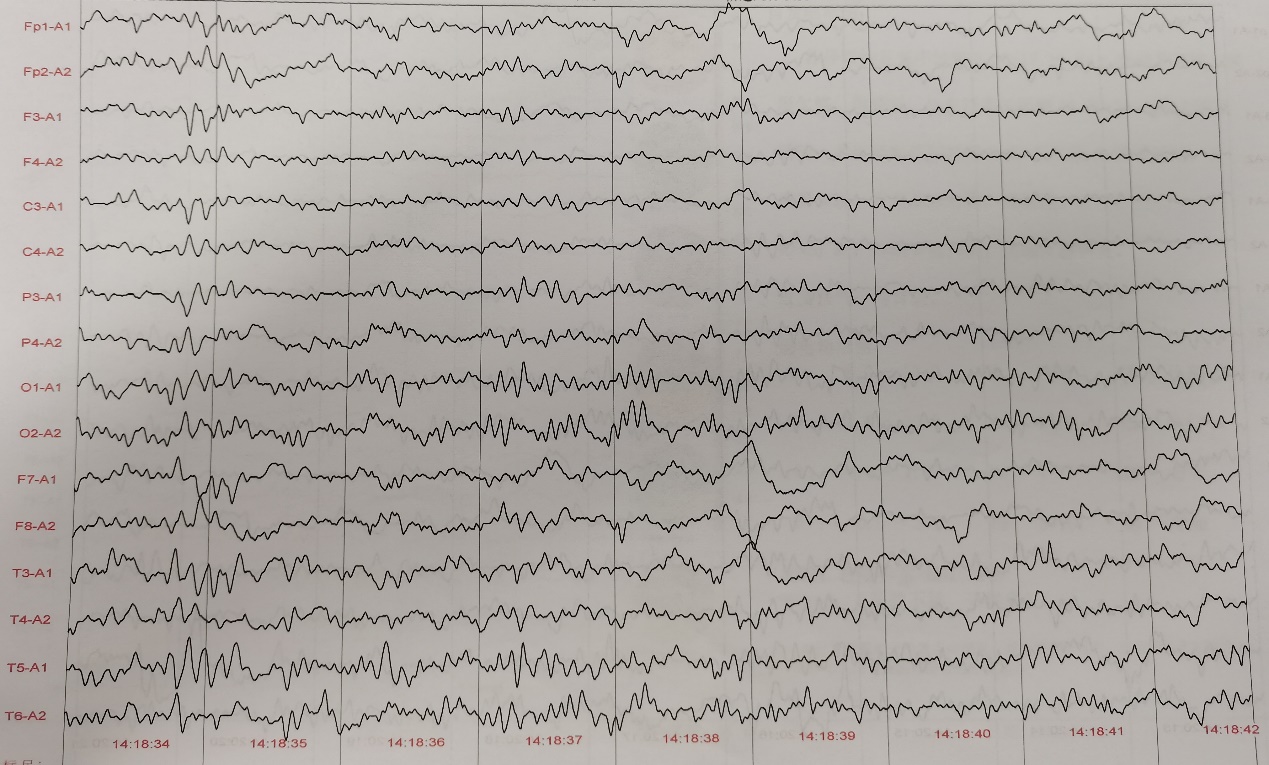


**Supplementary figure 1. Electroencephalogram evaluation of the patient.**

**Supplementary figure 2. Details of drugs treatment.**

*Annotation*: The patient’s symptoms still remained unchanged after escitalopram oxalate 10mg/d for nearly one month before hospitalization. In addition, this patient kept recovered after the ending of escitalopram oxalate.
